# Risk Factors Associated With Tuberculosis Infection Among Household Contacts of Patients With Microbiologically Confirmed Pulmonary Tuberculosis in 3 High Tuberculosis Burden Countries

**DOI:** 10.1093/infdis/jiaf320

**Published:** 2025-06-17

**Authors:** Thobani Ntshiqa, Jeniffer Nagudi, Yohhei Hamada, Andrew Copas, Stacie Stender, Issa Sabi, Elias Nyanda Ntinginya, Julieth Lalashowi, Manthomeng Matete, Keolebogile Ntshamane, Ithabeleng Morojele, Miyelani Ngobeni, Don Mudzengi, Lilian Tina Minja, Tobias Chirwa, Knut Lönnroth, Viola Dreyer, Stefan Niemann, Molebogeng Rangaka, Salome Charalambous, Kavindhran Velen

**Affiliations:** The Aurum Institute, Implementation Research Division, Johannesburg, South Africa; School of Public Health, University of the Witwatersrand, Johannesburg, South Africa; The Aurum Institute, Implementation Research Division, Johannesburg, South Africa; Institute for Global Health, University College London, London, United Kingdom; Institute for Global Health, University College London, London, United Kingdom; Jhpiego, Global Health Security, Baltimore, Maryland, USA; Mbeya Medical Research Centre, National Institute for Medical Research, Mbeya, Tanzania; Mbeya Medical Research Centre, National Institute for Medical Research, Mbeya, Tanzania; Mbeya Medical Research Centre, National Institute for Medical Research, Mbeya, Tanzania; Monitoring, Evaluation and Research Division, Jhpiego, Maseru, Lesotho; The Aurum Institute, Implementation Research Division, Johannesburg, South Africa; The Aurum Institute, Implementation Research Division, Johannesburg, South Africa; The Aurum Institute, Implementation Research Division, Johannesburg, South Africa; The Aurum Institute, Implementation Research Division, Johannesburg, South Africa; Université des Antilles, Institut de Systématique, Evolution, Biodiversité, Muséum National D’Histoire Naturelle, Paris, France; Mbeya Medical Research Centre, National Institute for Medical Research, Mbeya, Tanzania; School of Public Health, University of the Witwatersrand, Johannesburg, South Africa; Department of Global Public Health, Karolinska Institutet, Stockholm, Sweden; Molecular and Experimental Mycobacteriology, Leibniz Lung Center, Borstel, Germany; Molecular Epidemiology Department, German Center for Infection Research, Partner Site Hamburg-Lübeck-Borstel-Riems, Borstel, Germany; Université des Antilles, Institut de Systématique, Evolution, Biodiversité, Muséum National D’Histoire Naturelle, Paris, France; Molecular and Experimental Mycobacteriology, Leibniz Lung Center, Borstel, Germany; Molecular Epidemiology Department, German Center for Infection Research, Partner Site Hamburg-Lübeck-Borstel-Riems, Borstel, Germany; Institute for Global Health, University College London, London, United Kingdom; The Aurum Institute, Implementation Research Division, Johannesburg, South Africa; School of Public Health, University of the Witwatersrand, Johannesburg, South Africa; The Aurum Institute, Implementation Research Division, Johannesburg, South Africa; School of Public Health, University of the Witwatersrand, Johannesburg, South Africa

**Keywords:** tuberculosis infection, QuantiFERON-TB-Gold-Plus, household contacts, pulmonary tuberculosis, microbiologically confirmed

## Abstract

**Background:**

Although tuberculosis preventive therapy guidelines support testing for tuberculosis infection in household contacts (HHCs), this adds operational complexity and cost and has often been abandoned. To understand utility of testing, we determined prevalence and risk factors for tuberculosis infection and tuberculosis preventive therapy eligibility among HHCs.

**Methods:**

In a cross-sectional study conducted from July 2021 to September 2022 in Lesotho, South Africa, and Tanzania, we enrolled people with microbiologically confirmed pulmonary tuberculosis and their HHCs. HHCs were screened and tested for tuberculosis and tuberculosis infection using Xpert Ultra and QuantiFERON-TB-Gold-Plus, respectively. Generalized linear modelling was used to determine factors associated with tuberculosis infection, using robust standard errors. Tuberculosis preventive therapy eligibility was determined using World Health Organization criteria.

**Results:**

We enrolled 342 people with pulmonary tuberculosis and 964 HHCs: 61.9% (597/964) were female with a median age of 18 years (interquartile range, 8–39 years). Overall, tuberculosis prevalence was 3.4% (25/739; 95% confidence interval [CI], 2.2%–4.9%), while tuberculosis infection prevalence was 48.7% (348/714; 95% CI, 45.0%–52.5%). Having tuberculosis infection increased with age per year (adjusted odds ratio [aOR], 1.02; 95% CI, 1.01–1.03), being from Lesotho (aOR, 1.82; 95% CI, 1.04–3.20), previous tuberculosis history (aOR, 2.25; 95% CI, 1.05–4.79), and being HIV negative (aOR, 2.30; 95% CI, 1.31–4.04). Overall, 62.2% (518/833; 95% CI, 58.8%–65.5%) were eligible for tuberculosis preventive therapy.

**Conclusions:**

Almost half of tuberculosis-exposed HHCs aged ≥5 years had tuberculosis infection. Approximately two-thirds of HHCs were eligible for tuberculosis preventive therapy, implying that providing tuberculosis preventive therapy without prior testing for tuberculosis infection may be warranted in this population. Further work on cost-effectiveness is warranted when new tests become available.

**Clinical Trials Registration:**

ISRCTN10003903.

## Teaser

Almost half of tuberculosis-exposed household contacts had tuberculosis infection and two-thirds were eligible for tuberculosis preventive therapy, suggesting that providing tuberculosis preventive therapy without prior testing for tuberculosis infection may be warranted in high tuberculosis settings.

Approximately, 10.8 million people fell ill, and 1.25 million died of tuberculosis in 2023 [1]. Tuberculosis disease is preceded by tuberculosis infection, an asymptomatic state involving persistent immune response to stimulation by *Mycobacterium tuberculosis-*specific antigens with no evidence of active tuberculosis [2]. Tuberculosis infection prevalence is estimated to be around 5%–23% globally [3, 4], and 5%–10% of immunocompetent and ≥40% of immunocompromised persons may progress to active tuberculosis disease [3, 5, 6]. Sub-Saharan Africa is estimated to have the largest number of persons with tuberculosis infection, many of whom are also persons with human immunodeficiency virus (HIV), with Lesotho, South Africa, and Tanzania being among the top 10 countries with the highest HIV prevalence globally [7, 8].

Consequently, targeted interventions such as tuberculosis preventive therapy are recommended for those at risk of developing tuberculosis disease [9] in high tuberculosis and HIV settings, especially household contacts (HHCs) [10–13]. Evidence from clinical trials has shown that tuberculosis preventive therapy intervention benefits the most those with tuberculosis infection [14, 15]. Global targets set at the United Nations High Level Meeting in 2023 include providing tuberculosis preventive therapy to 45 million people by 2027, including 30 million HHCs of people with tuberculosis, and 15 million people with HIV (PWH) [9]. However, the uptake of tuberculosis preventive therapy remains very low among HHCs in high tuberculosis and HIV burden settings; one of the barriers cited has been the need for tuberculosis infection testing, which has introduced operational challenges for many settings with some countries opting to remove the need for tuberculosis infection testing [16].

Understanding the prevalence can inform the need for testing for tuberculosis infection in these populations. Limited data exists on tuberculosis infection prevalence estimates among HHCs using QuantiFERON-TB-Gold-Plus (QFT-Plus; Qiagen), an interferon-γ release assay (IGRA), which is often preferred over tuberculin skin test (TST) due to limited cross-reactivity with bacillus Calmette-Guérin (BCG) and ease of reading the test. Moreover, global trends have shown varying reductions in tuberculosis incidence between 2015 and 2023, with the World Health Organization (WHO) African Region reporting a 24% decline [1]. A decline in tuberculosis burden may have reduced the tuberculosis infection prevalence. Therefore, more recent data on tuberculosis infection prevalence is important to inform policy position on use of QFT-Plus testing in the context of tuberculosis preventive therapy scale-up. In addition, understanding risk factors for tuberculosis infection in this population group is also important to triage, prioritize, and optimize the delivery of tuberculosis preventive therapy strategies in high tuberculosis and HIV burden settings.

In this study, we determined the proportion of contacts who had tuberculosis infection, as well as factors associated with tuberculosis infection in Lesotho, South Africa, and Tanzania. We further determined the proportion of contacts who were eligible for tuberculosis preventive therapy among HHCs of people with microbiologically confirmed pulmonary tuberculosis in these 3 high tuberculosis burden countries.

## METHODS

### Study Design

We conducted a cross-sectional study from July 2021 to September 2022 as part of a larger project titled “Community and Universal Testing for tuberculosis among contacts (CUT-TB).” CUT-TB was a pragmatic cluster-randomized trial (ISRCTN10003903) conducted in 2 phases in Lesotho, South Africa, and Tanzania [17].

#### Study Setting

Data collection was conducted in the periurban communities within 3 Sub-Saharan countries, including Lesotho (Maseru, Thaba-Tseka, and Quthing districts), South Africa (Ekurhuleni district), and Tanzania (Songwe and Mbeya regions). The chosen study sites were situated in countries with varying background tuberculosis incidence rates, socioeconomic profiles, and differing HIV prevalence [1]. In 2023, Lesotho, South Africa, and Tanzania had an estimated tuberculosis incidence of 664, 427, and 183 per 100 000, respectively [1, 18–20].

#### Study Procedures

We recruited people with tuberculosis who were aged ≥18 years and diagnosed with drug-sensitive and/or drug-resistant tuberculosis within 6 weeks of being approached by the study team. Only those who had a positive smear, culture, and/or Xpert MTB/RIF Ultra (Cepheid) result were considered as having microbiologically confirmed pulmonary tuberculosis. Only people with tuberculosis who had at least 1 contact were enrolled. HHCs listed by each tuberculosis index patient were enumerated and recruited during household visits to participate in the study. A HHC was defined as any person who shared the same enclosed living space for 7 nights or for frequent or extended periods during the day with the index patient during the 3 months before commencement of the current treatment episode.

Among HHCs, a single sputum sample was collected for Xpert Ultra testing. HIV testing was offered to HHCs who had unknown HIV status or reported being HIV negative but had not had an HIV test undertaken within the past year. Blood samples were collected in a single visit for QFT-Plus testing from HHCs aged ≥5 years using a single lithium heparin tube. QFT-Plus processing, testing, and interpretation were done according to manufacturer's guidelines [21]. Those with positive QFT-Plus results—using manufacturer's recommended interferon-γ response threshold of ≥0.35 IU/mL in either TB1 or TB2 tubes—were considered tuberculosis infected. *M. tuberculosis* immunoreactivity, as demonstrated by a positive QFT-Plus result, was used as a proxy for *M. tuberculosis* infection—here in referred to as tuberculosis infection. One of the limitations for this definition is *M. tuberculosis* immunoreactivity can outlast tuberculosis infection elimination or clearance and positive IGRA response could indicate either recent or distal *M. tuberculosis* exposure in some population groups and settings [22].

### Study Outcome

This analysis focused on 2 main study outcomes: (1) the proportion of participants who had a positive QFT-Plus results, herein referred to as tuberculosis infection prevalence; and (2) the proportion of HHCs who were eligible for tuberculosis preventive therapy using WHO criteria—HHCs living with HIV, aged <5 years, and/or had tuberculosis infection. However, the age band for tuberculosis preventive therapy eligibility was <15 years in Lesotho.

### Data Management

REDCap (Research Electronic Data Capture. Paul Harris, REDCap, Vanderbilt University Medical Center, 1211 Medical Center Drive, Nashville, TN 37232) was used as a database for collection, storage, and management of data for CUT-TB study. An approved version of REDCap survey was administered by research assistants to collect data on demographic characteristics, socioeconomic status, and medical history.

### Statistical Methods

Basic descriptive statistics such as frequencies (n), percentages (%), measures of central tendency, and their corresponding measures of spread were used to summarize data. *χ*^2^ test or 2-sided Fisher exact test was used to compare categorical variables. Clustering at household level was taken into account for all estimates. To determine tuberculosis infection risk factors, we used generalized linear modelling using robust standard errors to account for clustering at household level with family binomial and link logit. Results were summarized using odds ratios (OR) and adjusted odds ratios (aOR) with their corresponding 95% confidence intervals (CI) and *P* values. Data were analyzed using Stata version 16 (Stata Corp).

### Ethical Considerations

The study received ethical clearance from Wits Human Research Ethics Committee (210107), Gauteng Health Research Ethics Committee, and Ekurhuleni District Health Ethics Committee (GP_202104_015) in South Africa; Johns Hopkins Bloomberg School of Public Health's Institutional Review Board (16967) and Lesotho National Health Research Ethics Committee (37-2021) in Lesotho; and Tanzania Medical Research Coordinating Committee (NIMR/HQ/R.8a/Vol.IX/3799) and Mbeya Medical Research and Ethics Review Committee (SZEC-2439/R.C/V.1/55). Prior to commencement of study procedures, we obtained informed consent from all study participants, their parents, or guardians using written informed consent and an information sheet available in the commonly used local languages and in accordance with ethical standards of the Helsinki Declaration.

## RESULTS

### Participants Enrolment

**Figure 1.**
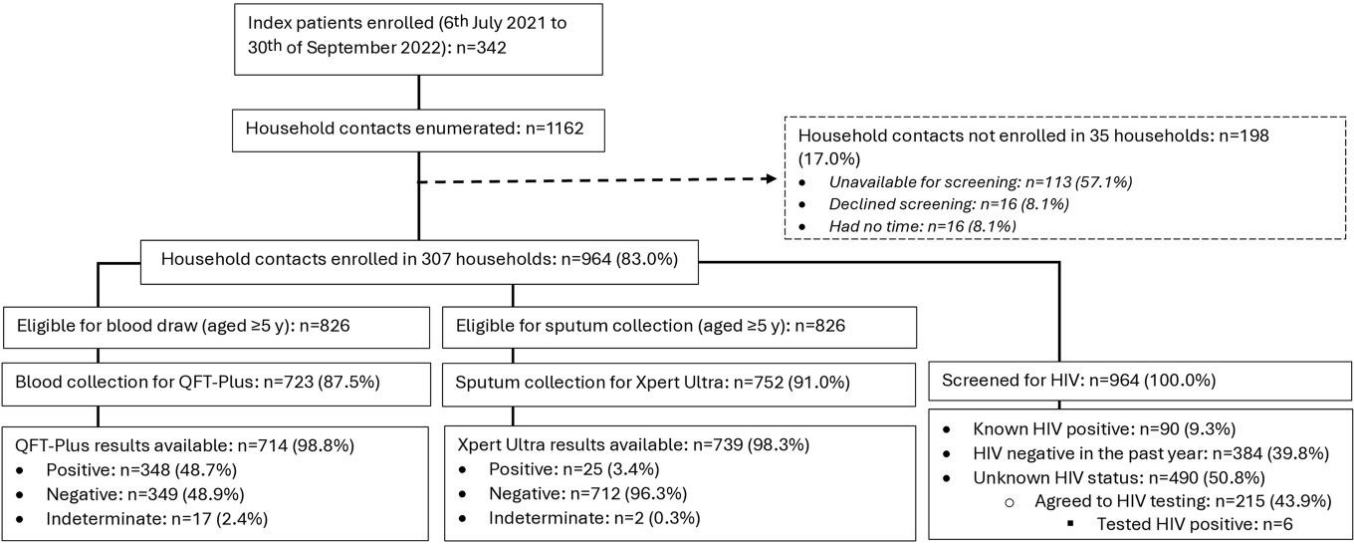
Participants flow chart at enrolment for tuberculosis index patients and their household contacts in 3 high tuberculosis burden countries. Abbreviations: HIV, human immunodeficiency virus; QFT-Plus, QuantiFERON-TB-Gold-Plus; Xpert Ultra, Xpert MTB/RIF Ultra.

A total of 342 people with tuberculosis were enrolled: 100 (29.2%) Lesotho, 152 (44.4%) South Africa, and 90 (26.3%) in Tanzania (Table 1). From these enrolled index patients, 1162 HHCs were enumerated, of whom 83.0% (964/1162) were enrolled from 307 (89.8%) households (Figure 1).

**Table 1.** Demographic Characteristics of Tuberculosis Index Patients and Their Household Contacts in 3 High Tuberculosis Burden Countries

Variable	Index Patients (n = 342)	Household Contacts (n = 964)
Median age, y (IQR)	39 (31–51)	18 (8–39)
Age category, y		
≤29	67 (19.6)	613 (63.6)
30–39	108 (31.6)	113 (11.7)
40–49	74 (21.6)	82 (8.5)
50–59	55 (16.1)	62 (6.4)
≥60	38 (11.1)	94 (9.8)
Sex		
Male	230 (67.3)	367 (38.1)
Female	112 (32.8)	597 (61.9)
Site country		
South Africa	152 (44.4)	300 (31.1)
Lesotho	100 (29.2)	321 (33.3)
Tanzania	90 (26.3)	343 (35.6)
HIV status		
Positive	159 (46.5)	96 (10.0)
Negative	171 (50.0)	593 (61.5)
Unknown	12 (3.5)	275 (28.5)
Sleep in the same room with index		
No	…	646 (67.0)
Yes	…	310 (32.2)
Missing	…	8 (0.8)
Previous tuberculosis		
No	…	909 (94.3)
Yes	…	51 (5.3)
Missing	…	4 (0.4)
Previous tuberculosis preventive therapy		
No	…	838 (86.9)
Yes	…	123 (12.8)
Missing	…	3 (0.3)

Data are No. (%) except where indicated.

Abbreviations: HIV, human immunodeficiency virus; IQR, interquartile range.

### Demographic Characteristics of Participants

Of the enrolled people with tuberculosis, 67.3% (230/342) were male with a median age of 39 years (IQR, 31–51 years) (Table 1). All participants had microbiologically confirmed pulmonary tuberculosis with 3.2% (11/342) being rifampicin resistant. Overall, 46.5% (159/342) of people with tuberculosis were PWH with 73.0% (116/159) being on antiretrovirals.

Of the enrolled HHCs, 61.9% (597/964) were female, and the median age was 18 years (IQR, 8–39 years). All HHCs were of black African ethnicity with 28.9% (279/964) sleeping in the same room as the tuberculosis index, 5.3% (51/964) had a history of previous tuberculosis treatment, and 86.9% (838/964) had no history of taking tuberculosis preventive therapy (Table 1).

### Tuberculosis Screening and Testing

Of the enrolled HHCs, 85.7% (826/964) were aged ≥5 years, 91.0% (752/826) produced a sputum sample for Xpert Ultra testing, and 98.3% (739/752) had results. Sputum quality was indicated in 641 HHCs, of which 61.8 (396/641) had good sputum quality. Overall, tuberculosis prevalence was 3.4% (25/739): 7.4% (11/147; 95% CI, 3.8%–12.9%) among symptomatic HHCs and 1.7% (14/816; 95% CI, .9%–2.9%) among asymptomatic HHCs.

### Prevalence of Tuberculosis Infection

#### Overall Tuberculosis Infection Prevalence

Of the enrolled HHCs, 85.7% (826/964) were eligible for blood draw (aged ≥5 years), of whom 87.5% (723/826) had blood drawn for tuberculosis infection evaluation using QFT-Plus and 98.8% (714/723) had QFT-Plus results. Of those with QFT-Plus results, 48.7% (348/714; 95% CI, 45.0%–52.5%) were QFT-Plus positive: 57.2% (123/215; 95% CI, 50.3%–63.9%) in Lesotho, 56.7% (135/238; 95% CI, 50.2%–63.1%) in South Africa, and 37.5% (90/240; 95% CI, 31.4%–44.0%) in Tanzania (Figure 1). Overall, 2.4% (17/714; 95% CI, 1.4%–3.8%) had indeterminate QFT-Plus results.

#### Tuberculosis Infection Prevalence by Demographic Characteristics

**Figure 2.**
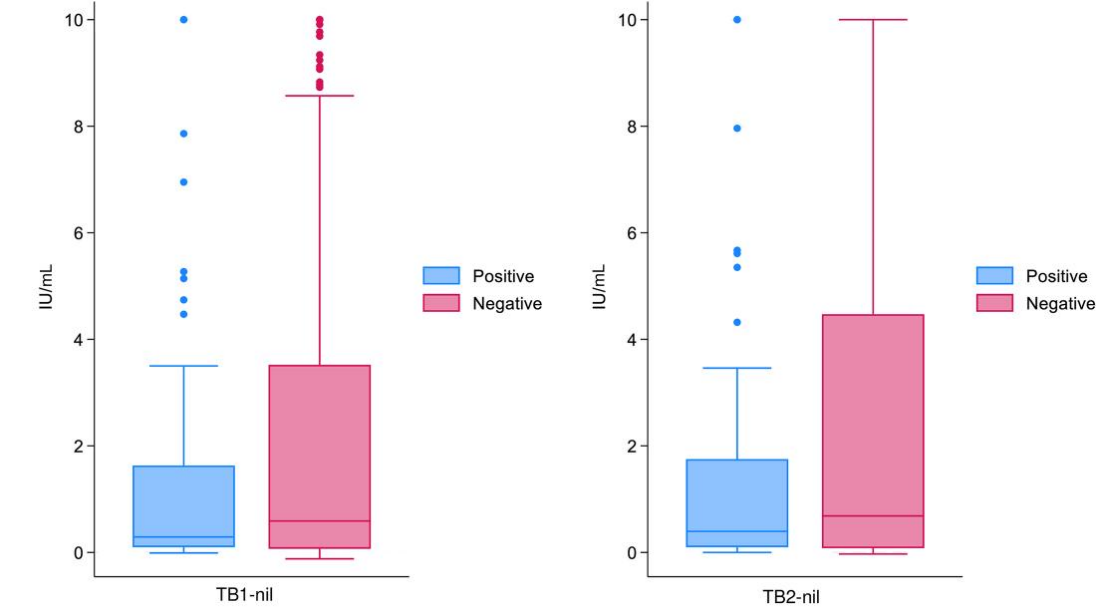
Box plot for interferon-γ response by HIV status among household contacts in 3 high tuberculosis burden countries.

In this analysis, 97.1% (693/714) of those with QFT-Plus results were included after excluding 21 HHCs (17 indeterminate QFT-Plus results and 4 missing data). The prevalence of tuberculosis infection was 49.9% (346/693; 95% CI, 46.1%–53.7%) (Table 2). HHCs who were aged ≥60 years (67.1%, 53/79; 95% CI, 55.6%–77.3%), or from Lesotho (56.3%, 121/215; 95% CI, 49.4%–63.0%) or South Africa (56.7%, 135/238; 95% CI, 50.2%–63.1%), had previous tuberculosis (73.8%, 31/42; 95% CI, 58.0%–86.1%), and those who were HIV negative (71.5%, 271/379; 95% CI, 66.7%–76.0%) were more likely to have tuberculosis infection. However, there was no significant difference in interferon-γ responses between HIV-negative and HIV-positive HHCs (TB1, z = −1.536, *P* value = .1246; and TB2, z = −1.732, *P* value = .0833; Figure 2). Among HHCs with rifampicin-resistant tuberculosis, the prevalence of tuberculosis infection was 62.5% (10/16; 95% CI, 35.4%–84.8%).

**Table 2.** Prevalence and Risk Factors for Tuberculosis Infection Among Household Contacts in 3 High Tuberculosis Burden Countries

Characteristics	No. (%)	QFT-Plus Positive, No. (%)	Univariable Analysis		Multivariable Analysis		
			OR (95% CI)	*P* Value	aOR (95% CI)	Local *P* Value	Global *P* Value
Household contacts (n = 693)							
No. (%)	693 (100.0)	346 (49.9)					
Median age, y (IQR)	26 (13–45)	32 (18–50)	1.03 (1.02–1.03)	<.001	1.02 (1.01–1.03)	<.001	<.001
Sex							.163
Male	255 (36.8)	129 (50.6)	1	Ref	1	Ref	
Female	438 (63.2)	217 (49.5)	0.96 (.71–1.30)	.790	0.78 (.55–1.11)	.163	
Site country							.109
Tanzania	240 (34.6)	90 (37.5)	1	Ref	1	Ref	
South Africa	238 (34.3)	135 (56.7)	2.18 (1.37–3.49)	.001	1.46 (.80–2.65)	.130	
Lesotho	215 (31.0)	121 (56.3)	2.15 (1.32–3.48)	.002	1.82 (1.04–3.20)	.036	
Sleep in the same room with index							
No	490 (71.2)	240 (49.0)	1	Ref	…	…	
Yes	198 (28.8)	103 (52.0)	1.13 (.78–1.63)	.517	…	…	
Previous tuberculosis							.036
No	649 (93.9)	315 (48.5)	1	Ref	1	Ref	
Yes	42 (6.1)	31 (73.8)	2.99 (1.48–6.03)	.002	2.25 (1.05–4.79)	.036	
Previous tuberculosis preventive therapy							
No	629 (90.9)	317 (50.4)	1	Ref	…	…	
Yes	63 (9.1)	29 (46.0)	0.84 (.49–1.45)	.528	…	…	
HHC HIV status							<.001
Positive	82 (11.8)	39 (47.6)	1	Ref	1	Ref	
Negative	379 (54.7)	271 (71.5)	1.48 (.89–2.45)	.130	2.30 (1.31–4.04)	.004	
Unknown	232 (33.5)	90 (38.8)	0.70 (.42–1.17)	.174	1.18 (.66–2.12)	.579	
Tuberculosis index patients (n = 284)							
Median age, y (IQR)	39 (31–51)	…	0.99 (.98–1.00)	.129	0.99 (.97–1.01)	.240	.240
Gender							.307
Male	198 (69.7)	…	1	Ref	1	Ref	
Female	86 (30.3)	…	1.25 (.82–1.90)	.306	1.28 (.79–2.08)	.307	
HIV status							.409
Positive	128 (45.1)	…	1	Ref	1	Ref	
Negative	146 (51.4)	…	1.27 (.86–1.87)	.230	1.32 (.87–2.01)	.190	
Unknown	10 (3.5)	…	1.67 (.53–5.21)	.379	1.34 (.39–4.52)	.642	
Smear result							
Positive	19 (6.7)	…	1	Ref	…	…	
1+ / 2+ / 3+	26 (9.2)	…	0.47 (.16–1.32)	.150	…	…	
Negative	6 (2.1)	…	1.37 (.28–6.77)	.701	…	…	
Unknown	233 (82.0)	…	0.68 (.30–1.53)	.348	…	…	
Merging smear results							
1+ / 2+ / 3+	45 (15.8)	…	1	Ref	…	…	
Negative	6 (2.1)	…	2.11 (.49–9.18)	.319	…	…	
Unknown	233 (82.0)	…	1.04 (.62–1.76)	.879	…	…	
Head of household							.385
Yes	145 (51.1)	…	1	Ref	1	Ref	
No	139 (48.9)	…	1.87 (1.26–2.75)	.002	1.24 (.76–2.02)	.385	
Index rifampicin resistance status							.543
Unknown	22 (7.7)	…	1	Ref	1	Ref	
RIF resistant	10 (3.5)	…	3.33 (.86–13.00)	.083	2.05 (.55–7.60)	.283	
RIF susceptible	252 (88.7)	…	2.09 (.92–4.73)	.079	1.47 (.62–3.50)	.383	
Number of people in household							.996
Median (IQR)	3 (1–4)	…	0.94 (.87–1.01)	.101	1.00 (.91–1.10)	.996	

Abbreviations: aOR, adjusted odds ratio; CI, confidence interval; HHC, household contact; HIV, human immunodeficiency virus; IQR, interquartile range; OR, odds ratio; QFT-Plus, QuantiFERON-TB-Gold-Plus; Ref, reference; RIF, rifampicin.

### Factors Associated With Tuberculosis Infection

In the multivariable analysis, HHC risk factors associated with tuberculosis infection were increasing age per year (aOR 1.02; 95% CI, 1.01–1.03; *P* < .001), being from Lesotho (aOR 1.82; 95% CI, 1.04–3.20; *P* = .036), previous tuberculosis history (aOR 2.25; 95% CI, 1.05–4.79; *P* = .036), and a negative HIV status (aOR 2.30; 95% CI, 1.31–4.04; *P* = .004) (Table 2). There were no index patient characteristics associated with tuberculosis infection.

### Tuberculosis Preventive Therapy Eligibility Among Household Contacts

**Figure 3.**
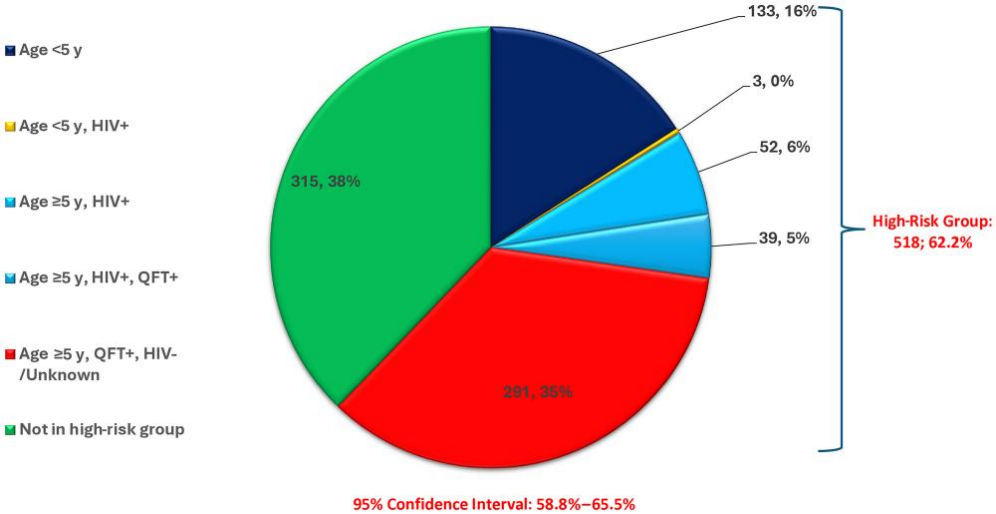
Tuberculosis preventive therapy eligibility characteristics for household contacts in 3 high tuberculosis burden countries (n = 833). Abbreviations: HIV, human immunodeficiency virus; QFT, QuantiFERON-TB-Gold-Plus.

**Figure 4.**
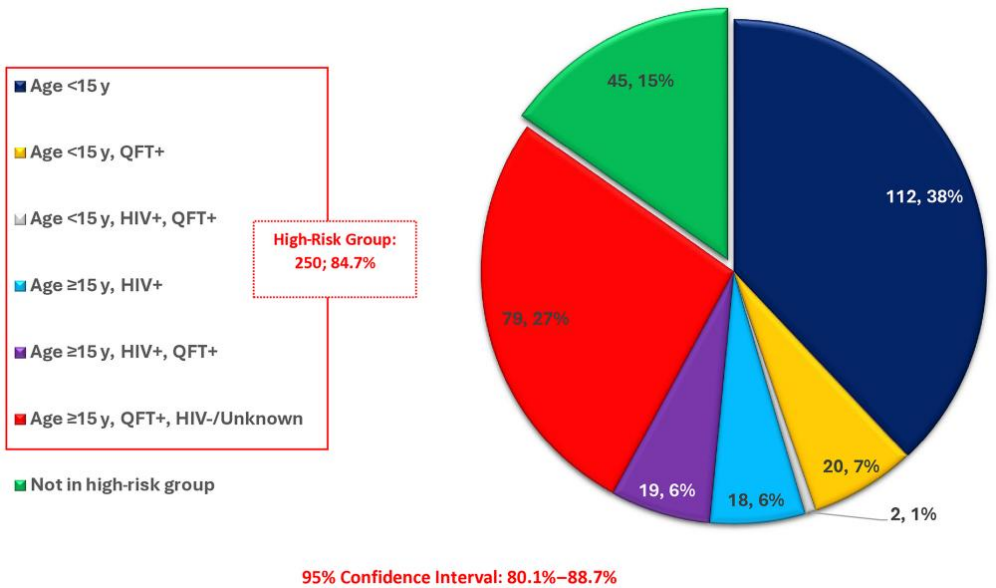
Tuberculosis preventive therapy eligibility characteristics for household contacts in Lesotho (n = 295). Abbreviations: HIV, human immunodeficiency virus; QFT, QuantiFERON-TB-Gold-Plus.

Overall, 10.0% (96/964; 95% CI, 8.1%–12.0%) of HHCs were PWH, of whom 93.8% (90/96; 95% CI, 86.9%–97.7%) were self-reported and 6.3% (6/96; 95% CI, 2.3%–13.1%) were diagnosed at the first household visit. The proportion of HHCs who were aged <5 years was 14.3% (138/964). Using WHO criteria for initiating tuberculosis preventive therapy, the proportion of HHCs eligible for tuberculosis preventive therapy was 62.2% (518/833; 95% CI, 58.8%–65.5%; Figure 3). By country, eligibility for tuberculosis preventive therapy was 68.6% (188/274; 95% CI, 62.8%–74.1%) in Lesotho, 63.9% (159/249; 95% CI, 57.6%–69.8%) in South Africa, and 55.2% (171/310; 95% CI, 49.4%–60.8%) in Tanzania (Table 3). However, when we applied Lesotho guidelines, which recommended tuberculosis preventive therapy for all contacts who are PWH, aged <15 years, and/or had tuberculosis infection, tuberculosis preventive therapy eligibility was 84.7% (250/295; 95% CI, 80.1%–88.7%) in Lesotho (Figure 4).

**Table 3.** Tuberculosis Preventive Therapy Eligibility by Country Among Household Contacts in 3 High Tuberculosis Burden Countries

HHC Eligibility Status	Lesotho (n = 274)			South Africa (n = 249)			Tanzania (n = 310)			Total (n = 833)			*P* Value
	No.	(%)	[95% CI]	No.	(%)	[95% CI]	No.	(%)	[95% CI]	No.	(%)	[95% CI]	
Eligible	188	(68.6)	[62.8–74.1]	159	(63.9)	[57.6–69.8]	171	(55.2)	[49.4–60.8]	518	(62.2)	[58.8–65.5]	.003
Not eligible	86	(31.4)	[25.9–37.2]	90	(36.1)	[30.2–42.4]	139	(44.8)	[39.2–50.6]	315	(37.8)	[34.5–41.2]	

Abbreviations: CI, confidence interval; HHC, household contact.

## DISCUSSION

The prevalence of tuberculosis infection among HHCs of people with tuberculosis was 48.7% as determined by a positive IGRA test. Age, study location, previous tuberculosis and HIV negative status were risk factors for tuberculosis infection. In addition, almost two-thirds of HHCs who were living with people with tuberculosis were eligible for tuberculosis preventive therapy in the 3 high tuberculosis burden countries using the WHO criteria for tuberculosis preventive therapy eligibility, comprising PWH, being aged <5 years, and having tuberculosis infection. The proportion of HHCs with microbiologically confirmed pulmonary tuberculosis was 3.4% as determined by a positive Xpert Ultra.

The tuberculosis infection prevalence found in this study is lower compared to prevalence reported in the PHOENIx trial (72%) among households of only patients with multidrug-resistant (MDR) tuberculosis [23]. However, this prevalence is in keeping with estimates from systematic reviews, which ranged between 42.4% and 51.5% among tuberculosis-exposed contacts [10, 13, 19]. This study provides the first estimate of tuberculosis infection burden among HHCs in Lesotho, while also providing updated estimates compared to previous studies in South Africa [24, 25] and Tanzania [26, 27]. We also found 62.6% of HHCs would have been eligible for tuberculosis preventive therapy. We further determined risk factors for tuberculosis infection to optimize the delivery of tuberculosis preventive therapy strategies among HHCs in high tuberculosis and HIV burden settings.

We observed a strong association between background tuberculosis prevalence and tuberculosis infection among HHCs in this study. The tuberculosis infection prevalence reported in Lesotho and South Africa in this study was much higher than the prevalence reported Tanzania, suggesting that risk of tuberculosis infection is higher in settings with a higher force of infection. In our study, we found familiar HHC risk factors associated with tuberculosis infection, including increasing age, being from Lesotho, previous tuberculosis history, and a negative HIV status.

The association between tuberculosis infection and age is consistent with findings from previous studies, including the cohort study conducted in South India [28], cross-sectional study conducted in rural KwaZulu-Natal, South Africa [29], and cross-sectional study conducted in Zambia and South Africa [30]. Some studies suggest that this association may be attributed to providing care to those who are sick with tuberculosis [28, 31] or a positive dose-response relationship [32, 33]. On the other hand, older HHCs are likely to have been infected in the past, prior to the current exposure, suggesting that tuberculosis infection is cumulative with age with little reversion. Therefore, it remains unclear whether caregivers and older HHCs may derive any benefit from targeted case finding efforts and provision of tuberculosis preventive therapy without prior testing for tuberculosis infection in high tuberculosis burden settings. In addition, tuberculosis preventive therapy may have less impact in contacts who were infected in the distant past. Because providing tuberculosis preventive therapy to older people may increase the risk of adverse events, the actual risk of disease would need to be carefully weighed against the expected rate of adverse events at specific ages.

The association between tuberculosis infection and previous history of tuberculosis is consistent with findings from previous studies conducted in Singapore [34], South Korea [35], and China [36]. Evidence from previous studies also shows that only a small proportion of patients with tuberculosis [37] or tuberculosis infection [38, 39] who receive treatment often revert from IGRA positive to negative after treatment completion, suggesting that IGRA may not be reliable surrogate marker of treatment efficacy or tuberculosis infection clearance. Provision of tuberculosis preventive therapy in this subpopulation can therefore not be based solely on tuberculosis infection status, but rather on comprehensive clinical assessment or algorithms.

The association between tuberculosis infection and a negative HIV status was observed in this study. However, this association might be reflective of loss of immune responsiveness in those with positive HIV status. Previous studies conducted in PWH found that the performance of IGRA can be affected by the immune status of a participant [40–42] or anergy [43–46], resulting in possible underestimation of tuberculosis infection burden. The low positivity on QFT-Plus testing among PWH and those with unknown status may reflect reduced test performance rather than low prevalence of tuberculosis infection.

We found that almost two-thirds of HHCs who were living with people with tuberculosis were eligible for tuberculosis preventive therapy in the 3 high tuberculosis burden countries using the WHO criteria for tuberculosis preventive therapy eligibility comprising PWH, being aged <5 years, and having tuberculosis infection. Therefore, providing tuberculosis preventive therapy to all HHCs seems a reasonable approach in the face of logistical complications and cost of tuberculosis infection testing. However, there is still a need to weigh the benefit of treatment against potential harm. We also found that 3.4% of HHCs had tuberculosis disease. This is consistent with recent systematic reviews, which reported a pooled prevalence of 3.3% to 3.6% among HHCs [10, 19, 47].

Overall, findings from our study are comparable to the findings from a multicountry feasibility study conducted among households of patients with MDR tuberculosis (PHOENIx trial) where 80% of HHCs were eligible for tuberculosis preventive therapy using the same combined WHO eligibility criteria [23]. Firstly, our study found a slightly lower proportion of HHCs who were eligible for tuberculosis preventive therapy, and this difference can be explained by the fact that in this study, only IGRA (QFT-Plus) was used to determine the tuberculosis infection status, while in the PHOENIx trial both IGRA and TST were used. Secondly, our study largely focused on drug-sensitive tuberculosis households while the PHOENIx trial enrolled patients with MDR tuberculosis; there are known differences in duration of infectiousness for these groups, which would influence the potential to transmit tuberculosis to their close contacts. Lastly, the proportion HHCs with unknown HIV status was higher in our study due to low testing uptake. Although we found a lower proportion of HHCs eligible for tuberculosis preventive therapy, this finding questions the need for tuberculosis infection testing prior to initiation of tuberculosis preventive therapy, as observed previously [14]. The proportion found to be positive in each country will help guide national programs as to the possible benefits and costs of including tuberculosis infection testing into the algorithm, especially as tests become more available, user-friendly, and potentially cheaper to administer. The high proportion of HHCs eligible for tuberculosis preventive therapy necessitates national policies focused on preventing progression to active tuberculosis through targeted tuberculosis preventive therapy.

Our study had several limitations. Firstly, the study was conducted in selected regions in each country with a relatively small sample size limiting the generalizability of our findings. Secondly, there was a low uptake of HIV testing services in this study, and this might have misclassified or underestimated tuberculosis preventive therapy eligibility among HHCs.

Thirdly, the use of a single tuberculosis infection test instead of 2 (ie, QFT-Plus and TST because there is no gold standard for tuberculosis infection testing), may have underestimated the prevalence of tuberculosis infection. However, previous studies found that the performance of QFT-Plus is not affected by prior BCG vaccination, unlike TST. In addition, tuberculosis preventive therapy eligibility assessment in this study did not evaluate other factors that also need to be taken into account before starting tuberculosis preventive therapy, such as recent completion of tuberculosis preventive therapy or medical contraindication. Lastly, there is currently no method to confirm whether a positive TST or IGRA result is due to recent exposure or exposure that occurred several years ago. Consequently, all positive results are considered recent infections, and all contacts are given tuberculosis preventive therapy although having tuberculosis infection may not necessarily translate to risk. This highlights an urgent need for better diagnostic tools that differentiate between those at high and low risk.

Our study adds to the growing body of literature on prevalence of tuberculosis infection among HHCs of people with tuberculosis in high tuberculosis and HIV settings. Some of the strengths of this study include the fact that this was a multicountry study. In addition, the study was designed by a multidisciplinary team with varying scientific expertise. Data collection was conducted in a systematic, meticulous, and standardized manner by experienced and trained field staff, resulting in high data quality.

## CONCLUSIONS

This study found that almost half of tuberculosis-exposed HHCs aged ≥5 years had tuberculosis infection. Age, study location, previous tuberculosis, and HIV negative status were risk factors for tuberculosis infection. The high prevalence of tuberculosis infection among HHCs reemphasizes the importance of this population for targeted case finding and prevention interventions, particularly in high tuberculosis burden settings. The study also found that almost two-thirds of HHCs who were exposed to people with tuberculosis were eligible for tuberculosis preventive therapy in the 3 high tuberculosis burden countries using the WHO criteria for tuberculosis preventive therapy eligibility, suggesting that putting those at risk of tuberculosis on tuberculosis preventive therapy without prior testing for tuberculosis infection may be warranted. However, further work on cost-effectiveness of tuberculosis infection testing should be considered to explore the utility of testing in high burden settings.
